# Catalytic Electron‐Driven Non‐Equilibrium Phase Transition in Quantum Electronic Heterostructures

**DOI:** 10.1002/advs.202507289

**Published:** 2025-10-30

**Authors:** Byung Cheol Park, Sahng‐Kyoon Jerng, Seung‐Hyun Chun, Hyeon Suk Shin, Fabian Rotermund, Bumki Min

**Affiliations:** ^1^ Sungkyunkwan University Suwon 16418 Republic of Korea; ^2^ Center for 2D Quantum Heterostructures Institute for Basic Science (IBS) Suwon 16419 Republic of Korea; ^3^ Department of Physics and Graphene Research Institute Sejong University Seoul 05006 Republic of Korea; ^4^ Department of Chemistry & Department of Energy Sungkyunkwan University (SKKU) Suwon 16419 Republic of Korea; ^5^ Department of Physics Korea Advanced Institute for Science and Technology (KAIST) Daejeon 34141 Republic of Korea

**Keywords:** catalytic electron, excitonic topological insulator, optical pump‐THz probe, phase transition, topological insulator

## Abstract

Thermodynamic phases of matter are defined by temperature, pressure, and particle number while their changes in solids are known to induce phase transitions. It is demonstrated that in heterostructures with an extra degree of freedom—two different electron pathways across the interface—electron flow creates new thermodynamic phases. Topological insulators (TIs) are used with two distinct electronic states: topological surface and confined insulating bulk states. The electrons can flow from a surface state to a bulk band (Path I) across the interface and vice versa (Path II). By manipulating electron density with light pulses and monitoring electron flow with ultrafast terahertz probes, a new thermodynamic phase of TI is identified with interfacial excitons (Phase II: excitonic TI), distinct from the equilibrium TI state (Phase I: TI). The results indicate that ≈3.5 × 10^12^ electrons (16% of the total) act as catalysts facilitating the transition to Phase II, while 84% (18.5 × 10^12^ electrons) occupy the new Phase II (excitonic TI). While the study focuses on ultrafast, pathway‐selective electron dynamics—beyond the conventional photodoping paradigm—the underlying principle of electron‐flow–mediated phase control through interface can be generalized to diverse quantum heterostructures for realizing emergent quantum states and for advancing quantum technologies.

## Graphical abstract

This study proposes an innovative method to control the phase of heterostructured materials via electron flow manipulation. Leveraging this technique, a new topological phase is achieved that hosts excitons at the interface of a topological insulator.

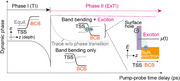

## 1 Introduction

According to the fundamental principles of thermodynamics, matter naturally evolves toward a state of minimal energy (U).^[^ ^1^, ^2^, ^3^ ^]^ This principle underlines the concept of the equilibrium phase of matter, the state with the lowest energy under specific thermodynamic conditions, such as temperature, pressure, and particle number. For instance, the internal energy (U) of an ideal gas in three dimensions is given by U = (3/2)Nk_B_T (in three dimensions), where N is the particle number, k_B_ is the Boltzmann constant, and T is the temperature.^[^ ^1^ ^]^ Changes in internal energy (dU) are proportional to the chemical potential (µ, the energy required to add a particle) and the change in particle number (dN), described by dU ∝ µdN.^[^ ^1^ ^]^ Consequently, the addition of particles into an ideal gas system is energetically unfavorable. (To avoid confusion, it is worth noting that the chemical potential µ itself also increases with increasing the particle number.)

We propose that electrons, analogous to an ideal gas, could significantly influence the thermal phases and properties of solids. This idea appears counterintuitive, as it challenges the conventional view that guest particles must conform to the constraints of the host. A fundamental question arises: Could the flow of electrons, rather than external factors, be the primary driver of a material's phase? This hypothesis can be investigated through methods that alter the material's intrinsic potential energy by controlling electron energy dU ∝ µdN via electron accumulation (e.g., through potential wells or electron localization due to correlations) or the control of electron flow.

By leveraging quantum electronic heterostructures as a platform, like topological insulator (TI),^[4‐6]^ the present work introduces the concept of electron flow‐driven phase transitions in solids. This idea is analogous to the recent discovery of exciton polarization‐induced ferroelectric transitions,^[^ ^7^ ^]^ which emphasizes the role of electronic components in driving phase transitions in materials. Specifically, we investigate how tailoring electron energy flow across heterostructure interfaces can trigger desired phase transitions. In heterostructures, a fraction of the electrons transfers energy (dU ∝ µdN), which facilitates the overcoming of energy barriers at the interface. These electrons act similarly to catalysts in chemical reactions, aiding phase transitions in solids. The remaining non‐catalytic electrons can then have opportunities to explore new phases of matter through electron‐induced phase transitions initiated by the catalytic electrons.

Although the potential landscape can be modified by applying a DC electric field, its steady state nature and limited potential reshaping hinder a complete understanding of the dynamic mechanisms underlying electron‐induced phase transitions. A more effective alternative involves dynamic approaches, such as ultrafast pump‐probe techniques. Ultrafast light irradiation (e.g., femtosecond laser pulses) can rapidly redistribute electrons within a material, while the subsequent probe captures the real‐time evolution of the phase transition process.^[^ ^2^, ^3^ ^]^ Therefore, while back‐gating (DC/AC) allows precise equilibrium tuning, optical excitation uniquely enables femtosecond‐scale access to non‐equilibrium pathways, transiently populating high‐energy states and revealing ultrafast phase transitions inaccessible via conventional gating. However, ultrafast measurements have limitations. It is challenging to achieve an ideal scenario where only electron redistribution occurs without perturbing the potential landscape of the sample. Despite this, we believe that careful control of experimental parameters, including pump fluence (incident photon energy per unit area, proportional to the number of excited electrons), time resolution, and a spectral range covering from infrared to ultraviolet, can adapt this technique to diverse materials and experimental conditions. These adjustments enable precise exploration of electron‐induced phase transitions.

The optical excitation of electrons into the conduction band places them in an unstable, high‐energy state.^[^ ^2^, ^3^ ^]^ Although these states are theoretically complex, they can be effectively described using a quasi‐equilibrium approach involving a chemical potential.^[^ ^2^, ^3^ ^]^ As a reliable parameter for thermodynamic description, the low‐energy transient conductivity (similar to the transient terahertz conductivity utilized in this study) accurately determines the chemical potential in this quasi‐equilibrium, which deviates from its equilibrium value.^[^ ^8^, ^9^ ^]^ Following excitation, the light‐excited system tries to return to a stable state either by transitioning into a new phase driven by dynamic electrons (both catalytic and non‐catalytic) or by returning to its initial phase. Importantly, the detection of the loss function,^[^ ^10^ ^]^ which reflects light‐induced electronic (longitudinal) polarization, provides clear evidence of inversion symmetry breaking. The sensitivity of the THz probe to this phenomenon consequently enables the observation of electron‐induced phase transitions.

## 2 Results and Discussions

**Figure 1:**
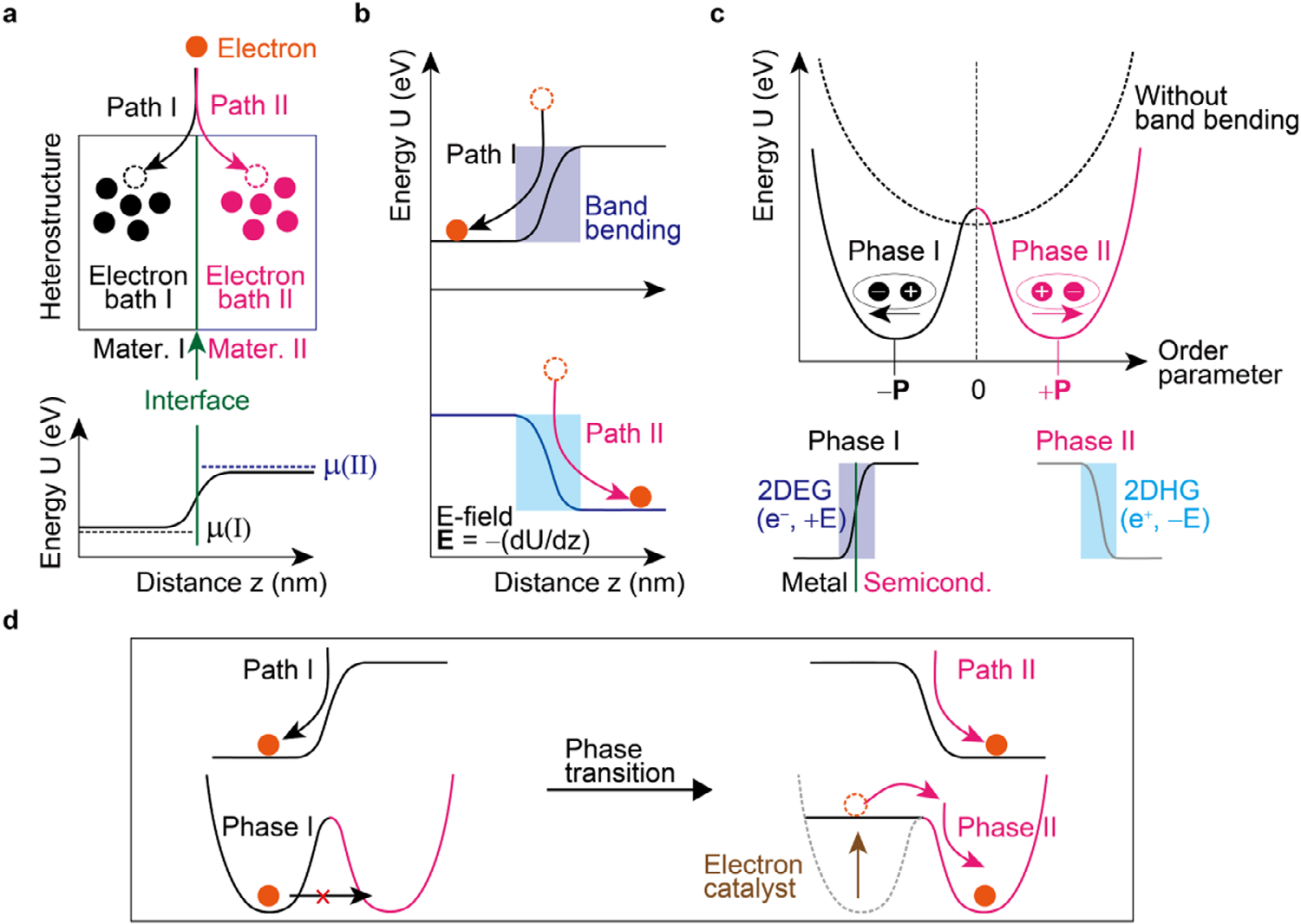
Schematic illustration of electronic heterostructure phases relying on the electron pathways. a) Schematic of a heterostructure composed of two materials (Mater. I & Mater. II) separated by an interface (green line and arrow). Electrons (orange circles) can be injected (outside the boxes) and follow two distinct pathways (Path I, black curly arrow; Path II, pink curly arrow). Filled circles within the boxes represent occupied electrons in their respective baths (Electron bath I & Electron bath II). Bottom panel describes the band bending (black line) due to different chemical potentials (µ(I); black dashed line & µ(II); blue dashed line). b) Spatial profile of the electronic potential, U(z), for the two pathways. The bending of the potential at the interface creates a strong electric field (**E**), whose direction depends on the chosen pathway. The shaded area illustrates the band bending region. c) Energy diagram. Without the band bending, the potential is parabolic (black dashed line). With the band bending, the electronic polarization (**P**) states are represented by a double‐well potential with two minima (black line for Phase I, pink line for Phase II). For a metal‐semiconductor junction, Phase I has 2D electron gas (2DEG; dark‐blue region) states with **P** antiparallel to the +z‐direction (bottom left) or 2D hole gas (2DHG; light‐blue region) with **P** parallel to the +z‐direction (bottom right). d) The figure illustrates a potential mechanism for a phase transition induced by electrons. (Left panel) The initial thermodynamic phase is represented, where a potential barrier prevents the system from transitioning to a new state. (Right panel) The introduction of electrons (namely, electron catalyst) can overcome this barrier, allowing the system to evolve into a new thermodynamic phase with a lower potential energy minimum.

Heterostructures provide a promising platform for electron‐induced phase transitions. Unlike bulk materials, heterostructures enable the manipulation of electron pathways, allowing electrons to follow the path either in Material I or Material II (**Figure** **1**a). The inherent potential difference between the two materials drives electron flow, pushing the system toward a new energy minimum (Figure 1b).

Heterostructure phases exhibit two distinct potential configurations determined by the sign of the chemical potential difference: a positive difference corresponding to Phase I, while a negative difference corresponds to Phase II. At the metal‐semiconductor interface (Schottky junction),^[^ ^11^, ^12^, ^13^ ^]^ this potential difference creates a strong electric field, inducing electronic polarization. Phase I corresponds to a 2D electron gas (2DEG), whereas Phase II represents a 2D hole gas (2DHG) (Figure 1c). What we want to report is the catalytic electron‐driven Phase II in topological insulators (TIs) with spatially‐indirect excitons at the electronic heterostructure boundary.

As electrons traverse the heterostructure interface, they interact with and modify the local potential landscape (Figure 1d). This dynamic interplay between the electrons and the potential landscape can trigger transitions to new stable (or metastable) phases representing temporary energy minima. Two distinct sets of electrons play crucial roles: one set acts as catalysts, modifying the potential landscape, while the other set enters new phase states, exploring new physical phenomena.

We utilize TIs with inherent heterostructures, which comprise a topological surface state (TSS) and a 2D electron gas state (2DEG) on an insulating bulk. The thin TSS and bulk (2DEG) states, each with unique quantum properties, form the electronic heterostructure when interfaced. Although band bending has been observed in TIs,^[^ ^12^, ^13^, ^14^ ^]^ its direct connection to the thermodynamic phase of the electronic heterostructure remains unclear. Since band bending induces electronic polarization (**P**), phase transitions may be linked to the exotic inversion‐symmetry‐broken topological state, as seen in polar TIs.^[^ ^15^, ^16^, ^17^ ^]^ The resulting non‐reciprocity is closely associated with the intriguing diode behavior.^[^ ^17^ ^]^ Moreover, as the strength of electric polarization (**P** ∝ E_0_ $\hat{z}$, where E_0_ is the applied electric field) influences Rashba splitting energies (H_R_ ∝ E_0_ $\left(\hat{z}\times \overset{⇀}{p}\right)\cdot \hat{\sigma }$, where $\overset{⇀}{p}$ is momentum and $\hat{\sigma }$ is the Pauli matrices),^[^ ^18^ ^]^ its control offers potential applications in spintronics.

**Figure 2:**
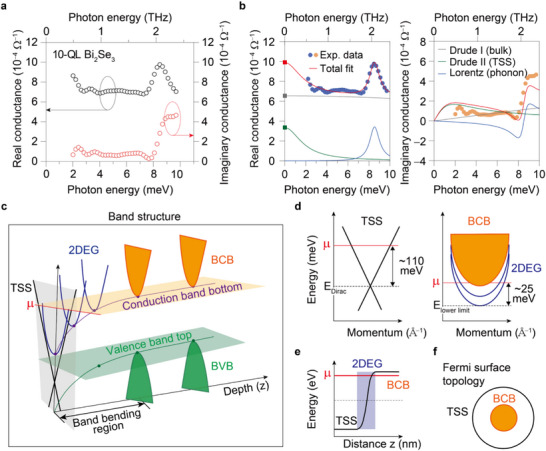
Equilibrium state of a topological insulator with Path I chosen by electrons. a) Equilibrium conductance of a 10‐quintuple layer (10 QL ≈10 nm thick) Bi_2_Se_3_ topological insulator with Path I chosen by electrons. The real part of the conductance is shown on the left *y*‐axis (black circles), and the imaginary part is on the right *y*‐axis (red circles). b) Analysis of the conductance using the Drude‐Lorentz model. This analysis extracts key physical parameters like chemical potential, carrier density, and scattering time. c) Schematic band diagram derived from our conductance analysis, consistent with conventional Bi_2_Se_3_ samples. Band bending is observed between the topological surface state (TSS; black) and the bulk band. BCB (orange) represents the bulk conduction band, while BVB (green) indicates the bulk valence band. This confinement creates a 2D electron gas (2DEG; blue) at the interface. µ represents the system's chemical potential. The electronic heterostructure is comprised of TSS and 2DEG, separated by their interface. d) Band diagram of individual TSS and 2DEG states obtained from the conductance analysis. E_Dirac_ marks the node of the TSS energy dispersion, and E_lower limit_ represents the bottom energy of the 2DEG. e, f) Band alignment (e) and Fermi surface topology (f) for the chosen electron pathway.

We selected TI, Bi_2_Se_3,_ with a thickness of ≈10 quintuple layers (QL) as a good candidate for the electronic heterostructure (the sample growth is detailed in Figure S1, Supporting Information and Experimental Section). This well‐established platform allows for comparable contributions of conductance from both TSS and bulk (**Figure** **2**a,b). At room temperature, the equilibrium conductance G_0_ is obtained from the raw transmittance of the THz field through algebraic transformation (see Experimental Section for conductance calculation and fitting analysis, and Table S1, Supporting Information for fitting parameters). Due to the thinness, the bulk contribution primarily arises from the 2DEG confined at the interface, as previously reported.^[^ ^12^, ^13^ ^]^ The minor bulk contribution is evident from the low conductance (G_0_ ≈1×10^−3^ Ω^−1^) of our 10‐QL Bi_2_Se_3_ film, which is ≈10 times smaller than the expected bulk conductance (≈1×10^−3^ Ω^−1^/ QL for doped Bi_2_Se_3_ film was reported in^[^ ^5^ ^]^).

Further analysis using the Drude‐Lorentz model (Figure 2b), commonly applied for low‐energy conductivity, helps extract key physical parameters of the electron system. These parameters include the plasma frequency, linked to the chemical potential, and the impurity scattering time associated with the broadening of the Drude peak (Table SI, Supporting Information). Our THz conductivity measurements reveal two distinct components: a sharp Drude peak and a broad Drude peak, signifying the presence of two types of charge carriers. While the THz conductance magnitude reflects intraband transitions near the Fermi level (on the order of meV) and is thus proportional to the density of states, its sharpness (i.e., scattering rate) is governed by the scattering mechanism, independent of band dispersion. Based on literature^[^ ^5^ ^]^ the sharp Drude peak corresponds to the conductivity of TSS carriers, characterized by significantly reduced backscattering from impurities. Conversely, the broad Drude peak is attributed to trivial bulk (2DEG) carriers. Although the bulk (2DEG) exhibits a Drude peak, it appears nearly constant within our measured range due to its broadness, but it does show frequency dependence.

Notably, the long impurity scattering time (equivalently, the low scattering rate) of electrons in the TSS, due to topological protection, allows us to distinguish the conductance of TSS from that of bulk (2DEG). The notation “bulk (2DEG)” is used to emphasize that the conductances of the bulk and 2DEG are generally indistinguishable. However, the distinction between TSS and bulk (2DEG) is evident in the Drude response, characterized by an absorption peak of free electrons centered at zero frequency– The sharper peak with a longer scattering time (~2.2 ps) corresponds to the TSS, while the broader peak with a shorter scattering time (~0.1 ps) reflects the bulk (2DEG) contribution (see Table SI, Supporting Information for fitting parameters). The topologically protected Dirac electrons are directly captured by the 1‐ps THz probe prior to impurity scattering, demonstrating the probe's ability to resolve ultrafast dynamics.

In addition to scattering time, the plasma frequency provides carrier densities for the TSS (n_TSS_ ≈3.34×10^12^ cm^−2^) and the bulk (2DEG) (n_bulk_ ≈2.46×10^19^ cm^−3^). Combining this data with the typical Bi_2_Se_3_ band structure, including the 2DEG at the interface (Figure 2c), allows us to determine the chemical potentials for the TSS (µ_TSS_ ≈106.6 meV) and the bulk (µ_bulk_ ≈25 meV) (Figure 2d). Additionally, this analysis reveals the band bending alignment and the Fermi surface topology (Figure 2e). This comprehensive analysis establishes a precise equilibrium state for our 10‐QL Bi_2_Se_3_ film, serving as a benchmark for investigating subsequent electron‐induced phase transitions.

**Figure 3:**
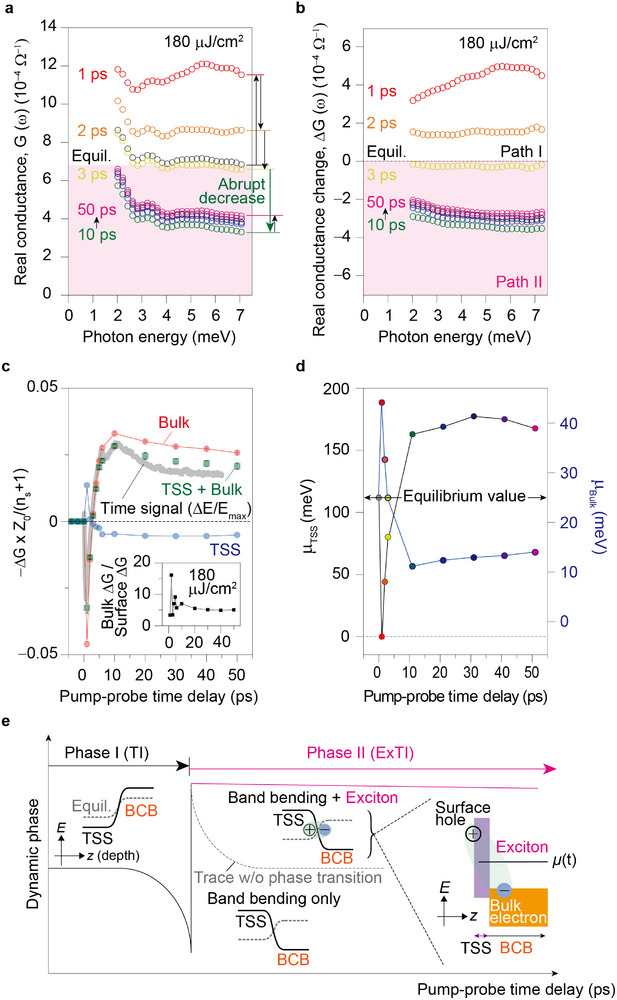
Transferring the electron pathway from Path I to Path II in a topological insulator by light. a, Real part of the transient conductance spectra (G(ω)) measured after an optical pulse with a fluence of 180 µJ cm^−^^2^. The equilibrium data (black circles) represent the conductance without optical excitation. b) Change in conductance (ΔG(ω)) compared to the equilibrium state. Path I dominates for the first few picoseconds (1‐3 ps), followed by an abrupt decrease and a transition to Path II (10–50 ps). c) Correlation between ΔG and the normalized time‐resolved signal (ΔE/E_max_). ΔG values are averaged over five data points ≈5.5 meV and 2.5 meV, respectively, for the 2DEG signal and TSS plus 2DEG signal (symbols). The TSS signal is extracted by subtracting the 2DEG signal from the combined signal. The observed dip in ΔE/E_max_ (gray line) near 7 ps likely originates from a replica of the strong dip at 1.4 ps, transmitted through the ≈430‐µm‐thick Al_2_O_3_ substrate. Here, Z_0_ = 377 Ω is the vacuum impedance and n_s_ = 3 is the refractive index of the substrate. d) The chemical potentials (µ) of the TSS (left *y*‐axis) and the bulk (right *y*‐axis) states. e) An overview of the dynamic phase transition in photo‐excited TI, encompassing both the excitonic TI (ExTI) and conventional TI phases. The formation of spatially‐indirect interfacial excitons is responsible for the extremely long response.

Our pump‐probe experiment unveils the ultrafast dynamics occurring within the inherent electronic heterostructure of Bi_2_Se_3_. The optical pump has a pulse duration of ≈60 fs and a repetition rate of 250 kHz (Reproducibility was also confirmed using a 1‐kHz system.). Despite the high signal‐to‐noise ratio, laser fluctuation can introduce variations in experimental data and extracted parameters. To mitigate these extrinsic effects, we performed multiple repeated measurements, using the average and standard deviation to determine our error bars. We use the bandwidth of the THz source to focus on the electronic responses, except phonon mode. **Figure** **3**a displays the transient conductance (G) captured at various time delays (τ) following photoexcitation, together with equilibrium conductance. The calculation of transient conductance follows the same procedure as the equilibrium case, with the THz transmittance modified by advanced optical pumping (Experimental Section). The analysis of transient conductance provides detailed insights into electron dynamics, uncovering significant time‐dependent variations as described below.

To describe the transient conductance, we assume a constant scattering time (and corresponding Drude peak broadening) during quasi‐equilibrium states, similar to the equilibrium condition. At 1 ps, the transient conductance is characterized by an increased, nearly constant bulk (2DEG) contribution and a decreased, sharp Drude‐like TSS component. By 2 ps, the TSS component recovers, but the elevated bulk contribution persists as a constant offset. At 3 ps, the system begins to return to its equilibrium conductance state. However, at 10 ps, a reversal occurs: the TSS conductance surpasses its equilibrium value while the bulk contribution decreases. This trend continues with minor adjustments toward equilibrium up to 50 ps. These conductance dynamics are further illustrated by the optical pump‐induced conductance change (ΔG) from the equilibrium conductance, shown in Figure 3b.

After τ ≈1 ps, the system reaches a quasi‐equilibrium state. In this regime, where thermodynamic descriptions and Drude analysis are valid (see Experimental Section), two crucial features emerge. First, the transient conductance (G) of the TSS initially dips but swiftly recovers before 3 ps while the bulk (2DEG) exhibits a significant rise that persists until ~1 ps before returning to its equilibrium value at 3 ps. In this initial dynamic phase, the TSS and bulk (2DEG) conductances exhibit inverse relationships, implying the electron flow from the TSS to the bulk (2DEG) across the interface. Later, the transient conductance of the TSS begins to increase, while that of the bulk (2DEG) shows a step‐like decrease after 10 ps.

While the initial quasi‐equilibrium dynamics (1 ps ≤ τ ≤ 3 ps) align with expectations, a surprising phenomenon emerges after 10 ps. Traditionally, energy relaxation is considered complete once the chemical potential returns to equilibrium, as seemingly reflected in our initial data. However, subsequent measurements reveal distinct behavior: a sharp decrease in bulk (2DEG) conductance after 10 ps (Figure 3a,b). Due to overlapping echo signals between 3 ps and 10 ps (refer to Figure 3c), this data range was excluded from analysis. Since such signals can overlap and overestimate transmittance changes, careful consideration is required for extracting dynamics, especially in regions where the signal sign changes. Consequently, subsequent overestimation of both relaxation time and conductance change in the long‐lived state due to echo superposition is possible. The lack of a significant external electron sink prevents further reduction of bulk conductance beyond its equilibrium value at 10 ps. The simultaneous increase in TSS conductance suggests a transfer of electrons from the bulk (2DEG) to the TSS. Importantly, the secondary dynamics after 10 ps indicate electron flow across the interface, but in the reversed direction compared to the initial dynamics.

**Figure 4:**
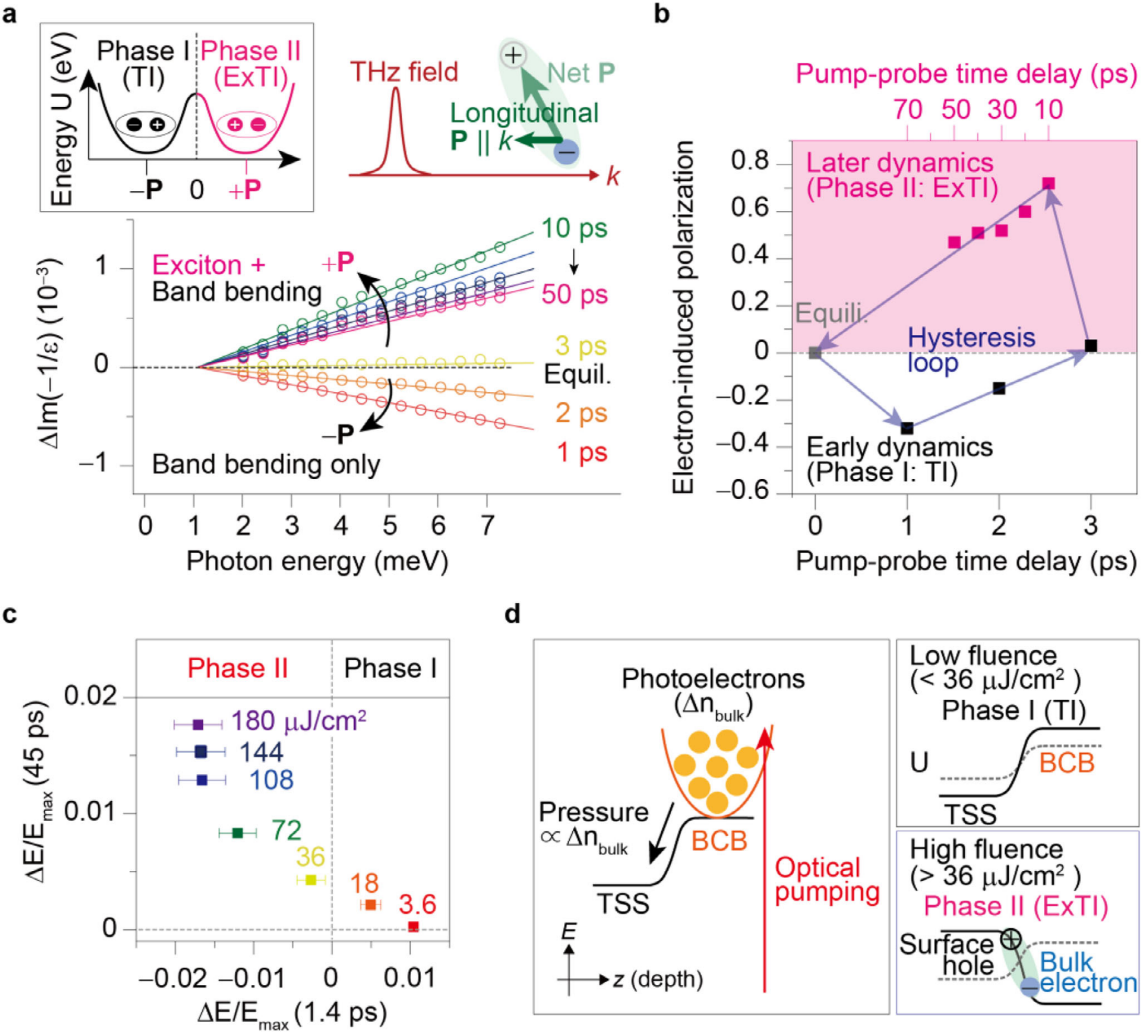
Phase transition toward Phase II is chosen by the electron. a) Dynamical change in the loss function (−1/ε) as a function of pump‐probe time delay. This indicates the longitudinal electronic polarization across the interface between TSS and bulk. The top figure presents the loss function measurement of interfacial excitons with a longitudinal polarization. The inset (box) illustrates the phase diagram involving two phases (Phase I with ‐P and Phase II with +P). b) The dynamical hysteresis, formed through the temporal evolution of the polarization. c) Distinguishing Phase I and Phase II: This panel demonstrates the presence or absence of Phase II based on the sign of ΔE/E_max_ at 1.4 ps. A negative ΔE/E_max_ (blue‐like dots) indicates dominant 2DEG character (Phase II), while a positive ΔE/E_max_ (red‐like dots) suggests dominant TSS character (absence of Phase II). Higher pump fluences promote Phase II (more blue dots). Data points represent averaged values over 3 ps with error bars showing standard deviation. d) Photoexcited electrons drive phase transition: The red arrow depicts optical pumping, generating photoexcited bulk electrons (yellow circles, Δn_bulk_). At low fluence (top), the band bending remains similar to the equilibrium state. However, at high fluence (bottom), the band bending flattens or even reverses due to the pressure exerted by the excess bulk electrons. Phase II is more stable in this scenario because the 2DEG, which counteracts the bulk‐TSS pressure, is absent.

To confirm the onset of this second dynamic phase, we analyze the time‐resolved signal (ΔE/E_max_, grey dots) depicted in Figure 3c. This signal represents the transmitted THz field remaining after absorption by both the TSS and bulk (2DEG). As a time‐domain signal, it averages the spectral responses of both components, capturing their collective dynamics. Our analysis reveals that the reversal indeed occurs at 3 ps, consistent with the results from the separate conductance analysis. While the skin effect, which typically describes a phenomenon in conducting samples where AC current flows primarily along the outer surface, is a recognized phenomenon in AC transport, becoming more significant at high (e.g., THz) frequencies, its impact is ruled out in our experiments. This is because our ultrathin film's thickness (<10 nm) is substantially below the relevant skin depth (>1 micrometer). However, for thicker bulk samples, this surface effect would require careful consideration in transient conductance analysis.

We further isolate the TSS and bulk (2DEG) contributions by comparing ΔE/E_max_ with ΔG within specific energy ranges. For quantitative analysis, ΔG was scaled by the vacuum impedance (Z_0_ = 377 Ω) and the substrate refractive index (n_s_ ≈3), as shown in Figure 3c. The red curve represents the 2DEG conductance change at 5.5 meV, while the green curve shows the combined TSS and 2DEG signal at 2.5 meV. The close agreement between ΔE/E_max_ and the 2.5 meV data (TSS plus bulk), in contrast to the deviation from the 5.5 meV data (bulk only). By subtracting the bulk‐only signal at 5.5 meV from the superimposed TSS and bulk signal at 2.5 meV, we successfully extract the TSS ΔG curve. Our measurements are performed in a time regime that can be treated as quasi‐equilibrium, not highly non‐equilibrium. This means the system largely retains its equilibrium Drude characteristics, allowing us to distinguish the two Drude components based on their scattering rates. Fortunately, the TSS Drude conductance exhibits a very sharp dispersion, effectively disappearing before 5.5 meV. This allows us to infer that carriers existing at or above 5.5 meV are almost entirely attributable to the bulk (including 2DEGs).

The thermodynamic phases of Bi_2_Se_3_ are clearly distinguished by a dynamical reversal in conductance changes (ΔG). Initially, ΔG is negative for the TSS and positive for the bulk, but these signs invert in the second response. The initial increase in bulk (2DEG) conductance (red curve) and decrease in TSS conductance (blue curve) suggest electron flow from the TSS to the bulk (Phase I). Conversely, after 10 ps (Phase II), the observed decrease in bulk conductance and increase in TSS conductance indicate a reversed electron flow, from the bulk to the TSS. It is important to note that manipulating the electron density with light pulses shifts the Fermi level relative to the band edges, thereby altering the ratio of surface to bulk conduction (inset of Figure 3c), whose saturation serves as a direct indicator of the phase transition.

This dynamic phase transition driven by the electron flow is reflected in the chemical potential (µ) of both the bulk (2DEG) and TSS states (Figure 3d). Drude analysis yielded the plasma frequency (ω_p_), directly correlated with electron density (n_e_) (see Experimental Section). The TSS chemical potential was calculated using µ_TSS_ = (hv_F_/2π^1/2^)n_TSS_^1/2^, where n_TSS_ is derived from the plasma frequency ω_p,TSS_ via spectral analysis (Experimental Section),^[^ ^5^, ^19^, ^20^ ^]^ representing the TSS electron density. The bulk chemical potential was determined by µ_bulk_ = (h^2^/2m^*^)(3π^2^n_bulk_)^2/3^ with n_bulk_ obtained from the plasma frequency ω_p,bulk_ (Experimental Section).^[^ ^5^, ^10^ ^]^ Our electron‐driven phase transition specifically refers to a change in the material's (or electronic structure's) phase that occurs when electrons flow across the interface of an electronic heterostructure after carrier injection. This is distinct from photo‐doping, where electron injection directly leads to a phase transition within a single electronic state (see Table S2, Supporting Information).^[^ ^21^ ^]^

In Phase I, the µ_TSS_ value decreases from 106.6 meV to nearly the charge neutral point (Dirac point), notably influenced by optical pumping (at 180 µJ cm^−^^2^). Conversely, the µ_bulk_ value increases from 25 to 40 meV, exhibiting an opposite trend to TSS conductance. In Phase II, µ_TSS_ increases up to 170 meV, while µ_bulk_ decreases to 15 meV at ~10 ps, subsequently undergoing a slow recovery toward the original value. However, equilibrium was not attained within our experimental timeframe, indicating a significantly longer lifetime for the second dynamics (after 10 ps), compared to the initial dynamics (1–3 ps). Although many‐body interactions may introduce minor deviations in conductance or chemical potential, our Drude‐model–based analysis reproduces the experimental data without distortion, indicating that such interactions do not significantly affect the interpretation.

Figure 3e summarizes the dynamical phase transition of a photo‐excited TI based on our experimental data. In equilibrium, the conduction band of the TSS meets the conduction band of the bulk at the interface in an n‐type TI, where no hole exists and thus exciton formation is prohibited. However, optical excitation induces band bending through a change in chemical potential. This leads to the formation of a p‐type TI where the hole band of the TSS meets the electron band of the bulk at the interface. Consequently, the photo‐excited holes in the TSS and electrons in the bulk can be Coulombically bound to form excitons spatially localized at the TSS‐bulk interface. This coexistence of a TI and excitons, which we term an “excitonic TI” (ExTI, distinct from an excitonic condensate), uniquely allows for the presence of excitons in a Dirac material. Remarkably, these spatially indirect interfacial excitons also exhibit long relaxation times due to hindered recombination. This finding highlights the significance of these excitons, analogous to momentum indirect excitons, and opens up new possibilities for the physics and applications of real‐space excitons. To our knowledge, there is a unique report of such spatially indirect excitons formed in a p‐type TI.^[^ ^22^ ^]^

Interestingly, we estimate the number of catalytic electrons required to induce the transition to Phase II (ExTI). Assuming photoexcited electrons recover within 1–3 ps, the total electron count in Phase II (ExTI) should match the equilibrium value. Any discrepancy between the equilibrium and 10 ps quasi‐equilibrium electron counts represents the catalytic electrons. Using the plasma frequency‐carrier density relationship (Experimental Section),^[^ ^5^, ^10^, ^19^ ^]^ we calculate carrier densities at 10 ps (early Phase II): n_TSS_ ≈1.04 × n_TSS_(equilibrium) and n_bulk_ ≈0.82 × n_bulk_(equilibrium). This yields total electron counts of N_total_(equilibrium) ≈2.20 × 10^13^ and N_total_(10 ps) ≈1.85 × 10^13^. A deficit of ΔN ≈3.5 × 10^12^ electrons is observed, indicating a violation of charge conservation, probably due to the loss as catalytic electrons. Therefore, we deduce that 3.5 × 10^12^ electrons (≈16% of total electrons) act as catalysts, while the remaining 18.5 × 10^12^ electrons (≈84% of total electrons) populate Phase II (ExTI) to form the spatially‐indirect interfacial excitons, considering the THz beam's interaction area through the circular aperture (A = πr^2^ = π(0.5 cm)^2^) and film dimensions (bulk thickness d = 10 nm, TSS depth δ ≈2.5 nm.^[^ ^5^, ^23^ ^]^ While the specific number of catalytic electrons may vary with doping level, their overall order of magnitude remains ≈10^12^. We note that, while interfacial excitons are present, their interaction with electrons is not substantial enough to preclude us from accurately describing the electrons using a single‐particle picture, as evidenced by the Drude peak in the excitonic TI regime.

Considering the early Phase II (ExTI), we can assume that there are negligible electron losses due to scattering or recombination. Indeed, numerous studies have reported extremely long recombination times for photo‐excited electrons in TIs,^[^ ^24^, ^25^ ^]^ and our high‐energy optical pump‐probe measurements also confirm that photoexcited electrons take a very long time (exceeding our measurement limit of 4 microseconds) to return to the valence band. Our experiments employed a thin TI film (10 QL layers thick). Unlike bulk crystals, this thin film geometry enables the recombination of photo‐excited carriers at the opposite surface after traversing the bulk material. Despite this increased potential for recombination in our thin samples, we observed a nearly persistent photo‐excited state that is comparable to that observed in thick bulk crystals. This observation strongly suggests that the mechanism responsible for the persistent photo‐excited state is intrinsic to the material properties and independent of sample thickness. Consequently, we propose that the formation of excitons at the interface provides a more plausible explanation for the persistent photo‐excited state than simple spatial separation of electrons and holes induced by band bending. Based on these findings, we hypothesize that a portion of the remaining ≈84% of photo‐excited electrons (excluding the catalytic electrons) form excitons, maintaining the inverted band bending (p‐type) state. These excitons gradually dissociate, restoring the original band bending (n‐type) state. The long‐lived spatially indirect interfacial excitons provide an excellent platform for realizing exciton‐driven ferroelectricity in two dimensions (i.e., exciton ferroelectrics.^[^ ^26^ ^]^)

The electron‐induced phase transition is corroborated by loss function analysis (Im(−1/ε), where ε is the complex dielectric function).^[^ ^10^ ^]^ This quantifies the longitudinal polarization, namely, the energy loss for a fast‐moving charged particle traversing a material along the light propagation direction (here, normal incidence). Derived from the algebraic relationship between conductivity (σ = G/d where d is the thickness) and permittivity ε (see Figure S2, Supporting Information for the calculation of permittivity ε),^[^ ^10^ ^]^ the loss function deviates from equilibrium, bifurcating into decreased values in Phase I and increased values in Phase II (**Figure** **4**a). The near‐zero loss function at 3 ps indicates negligible interfacial electron flow. These findings support a transition from a Phase I state with negative (longitudinal) polarization (−**P**) to a Phase II state with positive (longitudinal) polarization (+**P**), each residing in distinct energy minima of the potential diagram (Figure 4a inset). In Phase II (ExTI), it is difficult to accurately disentangle the contributions of band bending and excitons to the polarization, suggesting that the two effects are intertwined. The bifurcation and resulting hysteresis loop are evident in the dynamical evolution of the polarization as shown in Figure 4b, which reflects the phase transition caused by transferring electron pathways. Notably, the dynamics of ΔG (Figure 3c) and Δµ (Figure 3d) make it abundantly evident that the equilibrium and 3‐ps states are different even though they share the same zero polarization. Although the transient conductivity spectra exhibit some complexity, potentially involving excitonic contributions, these do not alter the primary conclusions of our study. Our analysis robustly attributes the long‐lived response to an ExTI state, ruling out other factors. Quantitative transient conductance measurements show the signal is solely explained by changes in Drude conductivity, not by phonons or other spectral components. The emergence of a longitudinal polarization in the loss function and a significant reduction in free carrier density (consistent with the sum rule) strongly support the formation of charge‐neutral interfacial excitons, confirming the ExTI state.

The enhanced magnitude of the loss function in Phase II (ExTI) compared to Phase I (TI) suggests a higher stability of Phase II (ExTI). This is mostly explained by the fact that Phase II contains interfacial excitons, which lower the potential energy. Additional minor contribution to the stability is the 2DEGs solely in Phase I that could act as a parasitic drain on the electron pressure toward the TSS. This could explain the longer relaxation time of Phase II (over 600 ps) relative to Phase I (only 3 ps). Notably, previous observations of electronic polarization in another TI, BiTeI, were linked to a broken inversion symmetry inducing intrinsic band bending.^[^ ^15^, ^16^ ^]^ In contrast, our Bi_2_Se_3_, lacking polar termination, exhibits polarization solely driven by light excitation, highlighting a unique mechanism. This implies a light‐induced, additional excitonic polarization rather than a modification of the original equilibrium band bending. We confirm that interfacial excitons can form even with a reversed band alignment compared to the reference work,^[^ ^22^ ^]^ thereby generalizing the concept of interfacial excitons in topological insulators. However, the electron and hole constituting the exciton possess reversed origins in our case (electron from bulk, hole from TSS), compared to Mori et al. (electron from TSS, hole from bulk). The observed loss function due to excitons appears as a relatively broad region rather than a single resonant peak, which is consistent with the presence of exciton complexes with binding energies in the few meV range, as discussed in the literature.^[^ ^27^ ^]^

To elucidate the mechanism underlying the electron‐induced phase transition in TIs, we correlate ΔE/E_max_ at 1.4 ps with ΔE/E_max_ at 45 ps (Figure 4c). This analysis reveals a causal link between dominant photoelectron type and the occurrence of Phase II (ExTI). A positive ΔE/E_max_ at 1.4 ps, indicative of TSS‐dominated photoelectrons, precludes the transition to Phase II. Conversely, a negative ΔE/E_max_ at 1.4 ps, signifying bulk‐dominated photoelectrons, facilitates the transition to Phase II. Increasing fluence leads to a greater ΔE/E_max_ at 45 ps (Phase II), implying a correlation between bulk photoelectron density (Δn_bulk_) and the magnitude of electron flow toward the TSS, akin to electron pressure (Figure 4d). At lower fluences (< 36 µJ cm^−^^2^), weak electron pressure results in minimal electron flow and subtle band bending modifications (Figure 4d, top right). In contrast, higher fluences induce substantial electron pressure, leading to significant band bending alterations, potentially even reversing the band bending (Figure 4d, bottom right).

**Figure 5:**
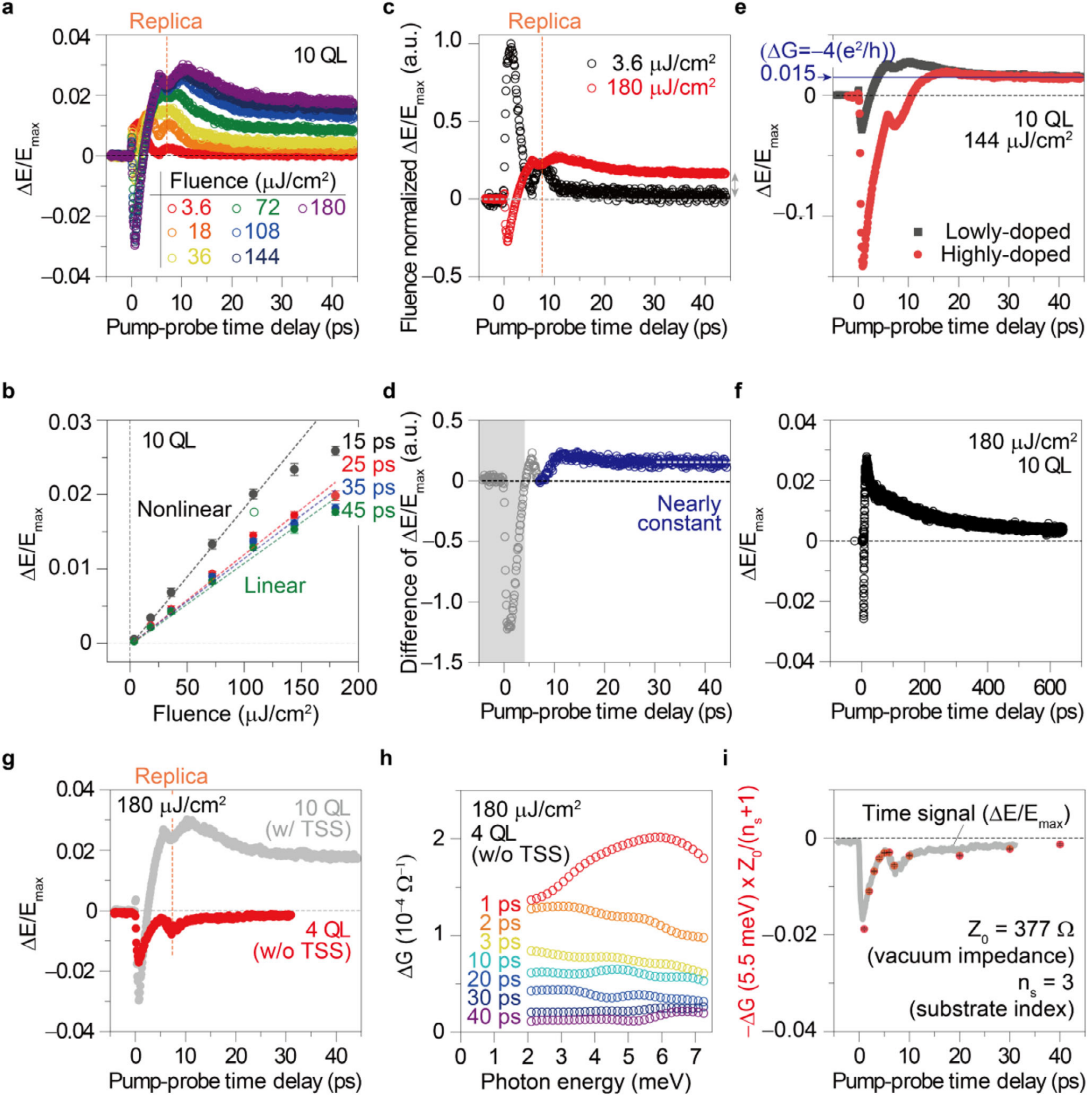
Characteristics of Phase II chosen by electron. a, b) Threshold fluence required for entering Phase II. Panel (a) shows the dependence of ΔE/E_max_ dynamics on fluence. Panel (b) presents ΔE/E_max_ values at specific times (15, 25, 35, and 45 ps). Linear dependence on fluence is observed at later times (25, 35, and 45 ps), unlike the non‐linear behavior at 15 ps, which is likely influenced by the replica signal. Data points represent averaged values over 3 ps with error bars indicating standard deviation. c) Clear visualization of the fluence‐dependent transition to Phase II (red, 180 µJ cm^−^^2^) compared to the low fluence case (black, 3.6 µJ cm^−^^2^). Each ΔE/E_max_ data is normalized by the corresponding fluence. d, The difference between the fluence‐normalized ΔE/E_max_ curves in (c). The second rise (blue dots) corresponds to Phase II dynamics, following the initial dynamics (gray dots) attributed to Phase I. e) Constant ΔE/E_max_ values for both highly and lowly‐doped 10‐QL samples after ~20 ps. This constant value (ΔE/E_max_ ≈0.015) corresponds to ΔG ≈−4(e^2^/h) in Phase II, implying the extraction of four quantum wells away from the chemical potential compared to Phase I. f) Delayed onset and extended duration over 600 ps. g, Topological surface state (TSS) necessity: An ultrathin Bi_2_Se_3_ film (4 quintuple layers, QL) lacking the TSS shows no Phase II (red dots, 4‐QL). The ΔE/E_max_ data for this film are compared to those of a 10‐QL sample (gray dots) with a well‐defined TSS, both excited with the same fluence. h) Absence of Phase II in ultrathin film: Consistent with the lack of TSS, the 4‐QL sample shows no negative ΔG (conductance change) within the first 40 ps. i) ΔE/E_max_ Reflects 2DEG Dynamics: For the 4‐QL sample with a TSS, ΔE/E_max_ (gray line) agrees well with ΔG (red dots) averaged ≈5.5 meV (2DEG contribution). This consistency suggests that ΔE/E_max_ primarily reflects the dynamics of the 2DEG, not the TSS. Details of the relationship are explained in the Experimental Section section.

Next, we turn to the characteristics of Phase II. We first reveal that the transition to Phase II occurs above the threshold fluence. In **Figure** **5**a, we plot the dependence of ΔE/E_max_ dynamics on fluence, showing the occurrence of the transition between 18 and 36 µJ cm^−^^2^. A similar threshold behavior is observed in another 10‐QL Bi_2_Se_3_ film with a comparable chemical potential level, confirming the reproducibility of our findings (see Figure S3, Supporting Information for a different sample data). We propose that at low fluences, the excitation of a limited number of electrons does not induce sufficient pressure to change the electric polarization. We also plot ΔE/E_max_ values at specific times (15, 25, 35, and 45 ps) in Figure 5b, showing a nearly linear fluence dependence at later times (25, 35, and 45 ps), unlike the non‐linear behavior at 15 ps, which is likely influenced by the replica signal (see Figure S4, Supporting Information for another example of the replica signal for graphene on sapphire substrate).

To clearly visualize the fluence‐dependent transition to Phase II (ExTI), we compare ΔE/E_max_ data at the maximum (180 µJ cm^−^^2^) and minimum (3.6 µJ cm^−^^2^) fluences used in our experiments. Normalizing ΔE/E_max_ data by fluence clearly distinguishes the two phases (Phase I at 3.6 µJ cm^−^^2^ and Phase II at 180 µJ cm^−^^2^) based on the presence of the secondary dynamics after 10 ps (Figure 5c). To isolate the second rise corresponding to Phase II (ExTI) dynamics, we calculate the difference between the fluence‐normalized ΔE/E_max_ curves (Figure 5d).

We observe a nearly constant ΔE/E_max_ from 10 to 40 ps during Phase II (ExTI). Figure 5e (black) presents ΔE/E_max_ for the lowly‐doped 10‐QL Bi_2_Se_3_ film (identical to the previously used sample, black squares). Intriguingly, the constant ΔE/E_max_ of 0.015 corresponds to a ΔG ≈−4e^2^/h in conductance quantum units, suggesting the removal of four quantum channels from the chemical potential. Unlike bulk conductance changes, this quantum behavior indicates the absence of four quantized 2DEG levels (two pairs of Rashba spin‐split 2DEGs)^[^ ^14^ ^]^ in Phase II (ExTI).

Notably, this phenomenon persists in highly doped 10‐QL films (see Figure S5, Supporting Information for the fluence dependent ΔE/E_max_ and conductance spectra). We perform the same experiment on a 10‐QL‐thick Bi_2_Se_3_ film with a relatively higher doping level (Figure 5e, red). As expected, the bulk signal with negative ΔE/E_max_ is significantly enhanced due to the higher doping. Interestingly, Phase II (ExTI) still occurs, but the dynamics begin slower than in the lowly‐doped sample. Our results indicate that higher doping levels impede the electron‐induced phase transition. We attribute this observation to the elevated equilibrium chemical potential in the highly‐doped system, which diminishes the relative impact of chemical potential changes under the same fluence. While the missing 2DEG states endure in Phase II (ExTI) for over 600 ps, a gradual recovery of the pulled 2DEGs to the chemical potential is evident from the slow decay of ΔE/E_max_ in Figure 5f. We further clarify that a significant reduction in the bulk (2DEG) ΔG / TSS ΔG ratio occurs when bulk (2DEG) G / TSS G ≈20 (Figure S6, Supporting Information), likely due to coupling between surface and bulk states, which is commonly observed in highly doped Bi_2_Se_3_ samples. By systematically tuning the surface‐to‐bulk electron density through chemical doping, we can directly manipulate electron pathways. THz conductivity measurements reveal that pathway selectivity depends on carrier density (or doping level).

To confirm the electronic heterostructure's critical role in the electron‐induced phase transition, we conduct additional thickness‐dependent measurements (Figure S7, Supporting Information), revealing that the electron‐pathway–driven phase transition vanishes below 5 QL—direct evidence that the interface is essential for governing the light‐induced phases in heterostructured topological materials. As the TSS in Bi_2_Se_3_ exhibits a depth profile of the wavefunction (δ ≈2.5 nm),^[^ ^5^, ^22^ ^]^ sub‐5 QL films experience hybridization between top and bottom TSS layers, resulting in the electronic gap. Therefore, the 4‐QL sample lacks a TSS capable of interacting with the THz field. Unlike the 10‐QL sample, this film displayed no positive ΔE/E_max_ transition after 30 ps, indicating the absence of Phase II (ExTI) (Figure 5g). The absent Phase II (ExTI) without the TSS (thereby, not establishing a heterostructure) confirms that the quantum interface plays a crucial role in electron‐driven phase transitions in electronic heterostructures. The bulk response dominated in the ΔG spectra throughout the pump‐probe delay τ, despite identical strong 180 µJ cm^−^^2^ pump fluence (Figure 5h). The time‐dependent ΔE/E_max_ signal is accurately described by solely bulk dynamics centered at 5.5 meV, as confirmed by the correlation between ΔE/E_max_ and −ΔG∙Z_0_/(n_s_+1) (Figure 5i). These findings underscore the necessity of a well‐defined interface for establishing electron pressure, a crucial factor in driving the electron‐induced phase transition. Eliminating one component of the heterostructure disrupts this interface, preventing electron pressure build‐up and inhibiting the phase transition.

Our findings demonstrate that electron flow across an ultrathin interface can induce phase transitions of the entire electronic heterostructure. This suggests that other degrees of freedom, such as spin, orbital, and phonon flow across the interface, could similarly trigger distinct (magnetic or structural) phase transitions, opening new avenues for exploring unconventional physical phenomena. While TIs under light‐excitation are excellent spintronic systems for generating spin currents, the formation of excitons at the interface in an excitonic TI could potentially reduce the practical spin current density. However, the unique combination of excitons with topology may enable the emergence of distinct quantum‐mechanical states, such as exciton condensates, even at room temperature. This has the potential to be a crucial breakthrough for the advancement of quantum technologies: The synergy of excitonic coherence and topological protection in our platform opens avenues for nonlinear optical devices,^[^ ^28^ ^]^ zero‐threshold lasing,^[^ ^29^ ^]^ and exciton bleaching and saturable absorption.^[^ ^30^ ^]^

The mechanism demonstrated here—pathway‐ and interface‐selective electron flow—can be extended beyond topological insulators to engineered quantum heterostructures, such as twisted bilayers and moiré superlattices. This approach opens up opportunities for applications in quantum information processing and low‐dissipation optoelectronics with ultrafast switching, which are inaccessible under equilibrium conditions using conventional electrical gating. In contrast to conventional photodoping, which induces phase changes solely by shifting the Fermi level, the observed exciton‐dressed topological phase emerges from selective electron flow across the surface–bulk interface. This topology‐enabled pathway selectivity reorganizes the electronic system into a new collective state, representing a mechanism fundamentally distinct from density‐driven transitions.

## 3 Experimental Section

### Bi_2_Se_3_ Ultrathin Film Growth

Bi_2_Se_3_ was grown by an ultra‐high high vacuum‐molecular beam epitaxy system, equipped with Knudsen effusion cells (Veeco). High purity bismuth (99.999+%) and selenium (99.999%) were supplied with the ratio of bi and se flux ≈1:15. The growth temperature was 250 °C, and post‐annealing temperature was 450 °C for the self‐crystallization (30 min) after the growth process. Detailed growth information could be found elsewhere.^[^ ^31^, ^32^ ^]^

### Optical Pump‐Terahertz Probe Experiments

We utilized our ultrafast measurement system based on Ti: sapphire femtosecond laser and regenerative amplifier operating at a repetition rate of 250 kHz (at KAIST). The measurements were also reproduced with the 1‐kHz system (at MPI‐P). THz probe pulses were generated from photo‐excited GaAs photoconductive antenna, while optical excitation pulses were directed onto the flat and shiny surface of the crystal. Their relative arrival time to the sample was controlled by varying their optical beam paths, initially separated after passing through the regenerative amplifier. Throughout the measurements, the pump beam spot size was maintained twice larger than that of the probe beam, ensuring that the THz probes scan only the laterally photoexcited region. The depth profile of the photoexcitation was determined by the penetration depth of the optical pump beam. Initially, the Bi_2_Se_3_ film was excited into a nonequilibrium state by optical pulses and then recovered to equilibrium through its intrinsic relaxation processes. The temporal dynamics of Bi_2_Se_3_ during the photoexcitation and relaxation were monitored in real‐time by the THz pulses, low‐energy electromagnetic fields in the energies in 1–10 meV.

### Conductance (G) Acquisition in (quasi‐)Equilibrium

We conducted our terahertz measurements, utilizing picosecond pulses in the time domain to acquire the complex transmittance through our Bi_2_Se_3_ films. We measured the time‐domain terahertz field transmitted through the sample (Bi_2_Se_3_ film on the sapphire substrate) without and with the optical pumping (λ ≈800 nm) for equilibrium and quasi‐equilibrium, respectively. Normalization against the substrate response is achieved by a separate measurement on a blank substrate. Time‐domain signals are then Fourier‐transformed to generate frequency‐dependent spectral functions. The normalized complex transmittance is then converted into the complex conductance spectra based on Tinkham's formula, valid in the thin film limit as studied here. The formula is given by t_fs_/t_s_ = 1/[1+Z_0_G/(n_s_+1)] where t_fs_ and t_s_ are the complex transmission coefficients of the sample (film plus substrate) and the bare substrate, respectively, while Z_0_ is the vacuum impedance, G the conductance of the film, and n_s_ the refractive index of the bare substrate. For bulk free‐carrier states, G = σd , where σ is the optical conductivity and d is the thickness of the film.

### Conductance Analysis with Drude‐Lorentz Model

We exploited the Drude‐Lorentz model for the conductance analysis in Bi_2_Se_3_ film. In the present case, the free carriers in the TSS and bulk (2DEG) directly couple with the terahertz radiation and exhibit distinctive absorption features within our experimental frequency range.

We analyze the THz conductivity using the Drude model, justified because (i) the optical pump spot is much larger than the probe, producing a nearly uniform carrier distribution; (ii) the conductivity is dominated by intraband momentum scattering, as widely adopted in ultrafast THz studies^[^ ^3^ ^]^ and (iii) diffusion is negligible on the sub‐10 ps timescale of our measurements.

Both equilibrium and quasi‐equilibrium states exhibit distinct Drude peaks for the TSS and bulk (2DEG), as described below:^[^ ^5^ ^]^

(1) $$G_{\mathrm{electron}}\left(\omega \right)=\frac{i}{4\pi }\;\frac{\omega_{p,\mathrm{TSS}}^{2}}{\omega +i\gamma_{\mathrm{TSS}}}\left(2\delta \right)+\frac{i}{4\pi }\frac{\omega_{p,\mathrm{bulk}(2\mathrm{DEG})}^{2}}{\omega +i\gamma_{\mathrm{bulk}(2\mathrm{DEG})}}d$$

where ω_p_ is the plasma frequency, γ (= 1/τ) is the scattering rate (= the inverse impurity scattering time), and δ (≈2.5 nm) is the depth of the TSS wavefunction.

In the equilibrium state, we incorporate phonon absorption (specifically, α‐phonons) within the Bi_2_Se_3_ film, resulting in a Lorentzian resonance as described below:

(2) $$G_{\mathrm{Phonon}}\left(\omega \right)=\frac{i}{4\pi }\frac{\Omega_{p}^{2}\omega }{\omega^{2}-\Omega_{0}^{2}+i\Gamma \omega }d$$

where Ω_p_ is the oscillator strength, Ω_0_ is the center frequency, and Γ is the broadening parameter. To isolate electronic contributions, we restricted the terahertz source's spectral range, thereby minimizing phonon absorption in the quasi‐equilibrium state.

### Carrier Density and Chemical Potential Calculation from Plasma Frequency

Carrier density, and consequently chemical potential, is directly linked to plasma frequency within each conduction channel. To determine these parameters, we applied distinct calculation methods to the individual channels, as detailed below:

For TSSs (two TSSs from the top and bottom of the sample), the plasma frequency ω_p_ is proportional to n_TSS_^1/4^, as the 2D linearly dispersed band.^[^ ^19^, ^20^ ^]^

(3) $$\omega_{p,\mathrm{TSS}}^{2}\cdot 2\delta =\left(1/60\pi \right)\left(v_{F}e^{2}/4\hbar \right)\sqrt{\pi \left|n_{\mathrm{TSS}}\right|}$$

where v_F_ is the Fermi velocity (here, estimated to 1.0 × 10^6^ m s^−1^), e is the elementary charge, and ℏ (= h/2π) is the reduced Planck's constant. Also, the chemical potential is proportional to n_TSS_^1/2^:

(4) $$\mu_{\mathrm{TSS}}=\hbar \mathrm{vF}\sqrt{\pi n_{\mathrm{TSS}}}$$

On the other hand, for bulk (2DEG), the plasma frequency ω_p_ is proportional to n_bulk(2DEG)_^1/2^, as the conventional 3D parabolic band.^[^ ^10^ ^]^

(5) $$\omega_{p,\mathrm{bulk}(2\mathrm{DEG})}^{2}\cdot d=\left(4\pi n_{\mathrm{bulk}\left(2\mathrm{DEG}\right)}e^{2}/m^{*}\right)\cdot d$$

where m* (≈0.45 m_e_) is the effective mass of the bulk band. Then, the chemical potential is proportional to n_TSS_^2/3^:

(6) $$\mu_{\mathrm{bulk}(2\mathrm{DEG})}=\left(h^{2}/2m^{*}\right){\left(3\pi^{2}n_{\mathrm{bulk}(2\mathrm{DEG})}\right)}^{2/3}$$

where h is the Planck's constant.

### Light‐Induced Conductance Change (ΔG) Calculation

The conductance change ΔG(ω) of Bi_2_Se_3_ is also compared with the approximation methods below. The pump‐induced conductance change ΔG(ω) = G(ω) − G_0_(ω)—where G_0_(ω) and G (ω) are the complex conductance with and without the optical pump, respectively—is calculated from the transmittance ratio,ΔT(ω) = T(ω) − T_0_(ω), where T(ω) and T_0_(ω) are the complex transmission with and without the optical pump, respectively:^[^ ^33^ ^]^

(7) $$\frac{\Delta T(\omega )}{T(\omega )}=\frac{1+\left[G_{0}\left(\omega \right)Z_{0}\right]/\left[n_{s}+1\right]}{1+\left[G\left(\omega \right)Z_{0}\right]/\left[n_{s}+1\right]}\;-1\approx -\Delta G\left(\omega \right)\frac{Z_{0}}{n_{s}+1}$$

where Z_0_ (= 377 Ω) is vacuum impedance and n_s_ (≈3) is the refractive index of the sapphire substrate. Herein, two approximations are applied: first, the substrate index under the illumination of the optical pump beam remains constant; second, based on the thin‐film approximation, the denominator factor G(ω)Z_0_ is assumed to be much smaller than 1, i.e., G(ω)Z_0_ ≪ 1.

## Conflict of Interest

The authors declare no conflict of interest.

## Author Contributions

B.C.P. and B.M. conceived this work. S.‐K.J. and S.‐H.C. grew the high‐quality Bi_2_Se_3_ epitaxial films. B.C.P. measured and analyzed optical pump‐THz probe data, with the experimental support from both F.R. at KAIST and M.B. at MIP‐P. B.C.P., H.S.S., F.R., and B.M. wrote the manuscript with input from other co‐authors.

## Supporting information



Supporting Information

## Data Availability

The data that support the findings of this study are available from the corresponding author upon reasonable request.
