# Supplementary material for: Catalytic Electron‐Driven Non‐Equilibrium Phase Transition in Quantum Electronic Heterostructures
Source: Adv Sci (Weinh). 2025 Oct 30;13(3):e07289. doi: 10.1002/advs.202507289 (PMC12806539; doi:10.1002/advs.202507289)
Supplement: Supplementary file 1 — Supporting Information [file ADVS-13-e07289-s001.docx]

**
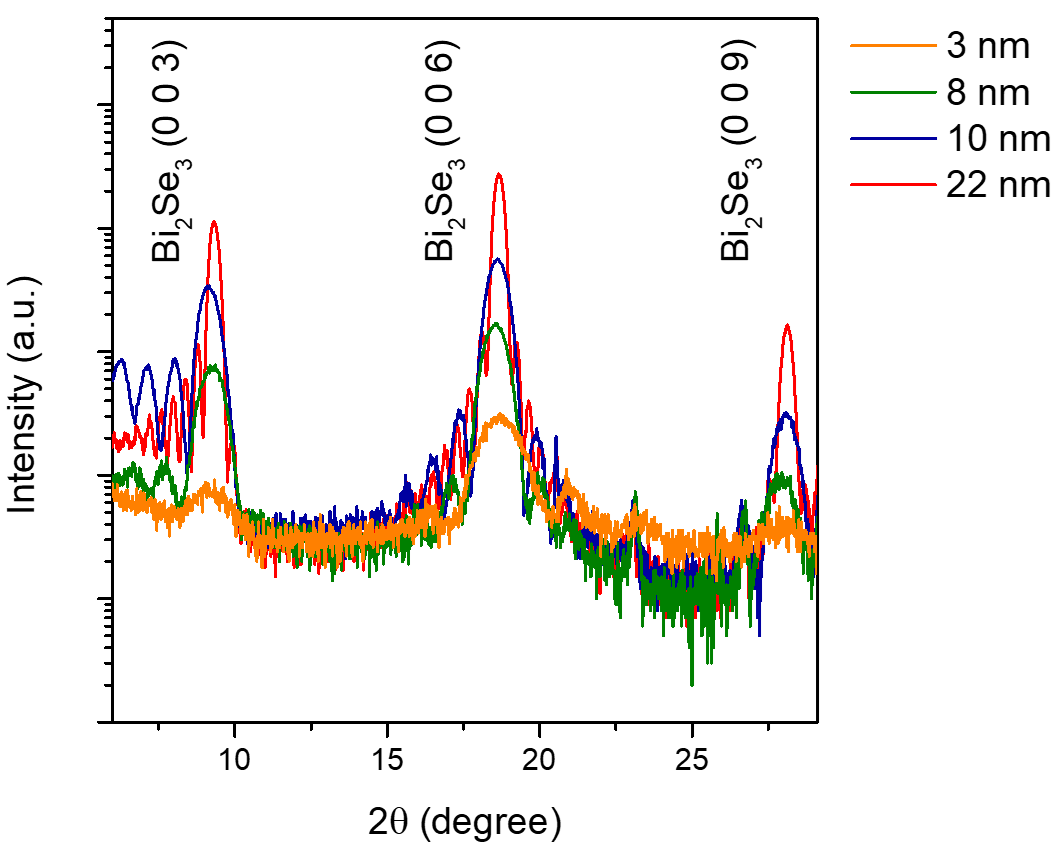
****Supporting Fig. S1 | The XRD patterns of our MBE-grown Bi_2_Se_3_ were obtained by X-ray diffractometer (Rigaku D/MAX-2500).** Cu K_α_ radiation (wavelength of 1.54 Å) was used to investigate crystalline structures. The XRD profile shows the out-of-plane ordered Bi_2_Se_3_ with (003) families of *R3-m* structure.

**
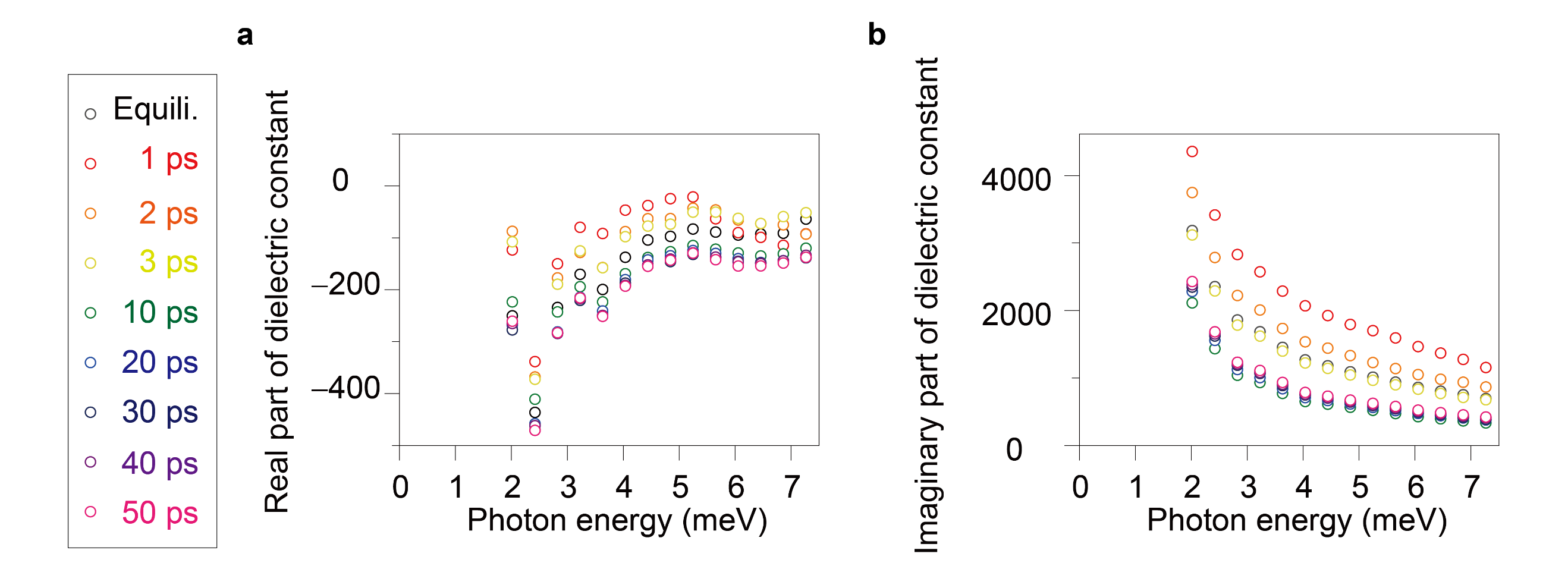
Supporting Fig. S2. Dielectric constant ε spectra of the 10-QL Bi_2_Se_3_ film (identical sample mainly used in the present work).**

**
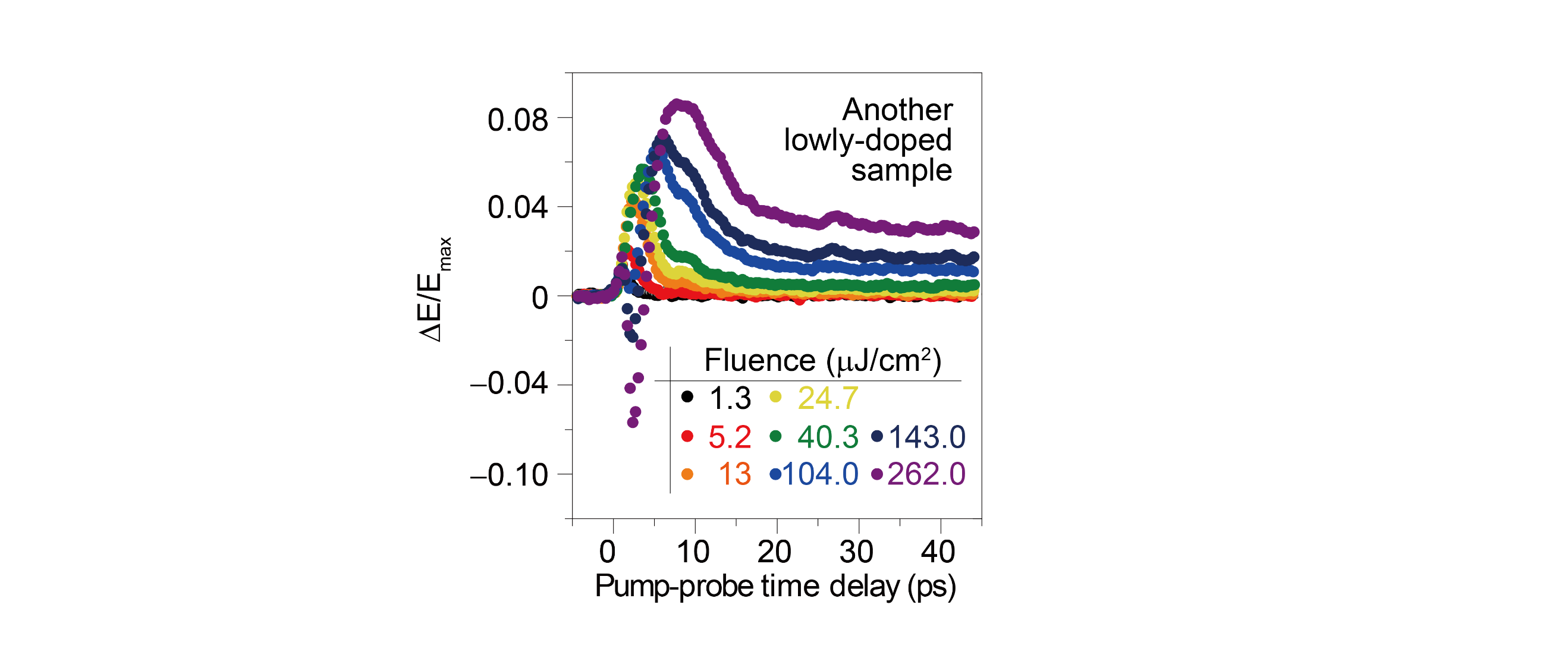
**

**Supporting Fig. S3. Optical pump-terahertz probe experiments for another lowly-doped 10-QL Bi_2_Se_3_ film.**

**
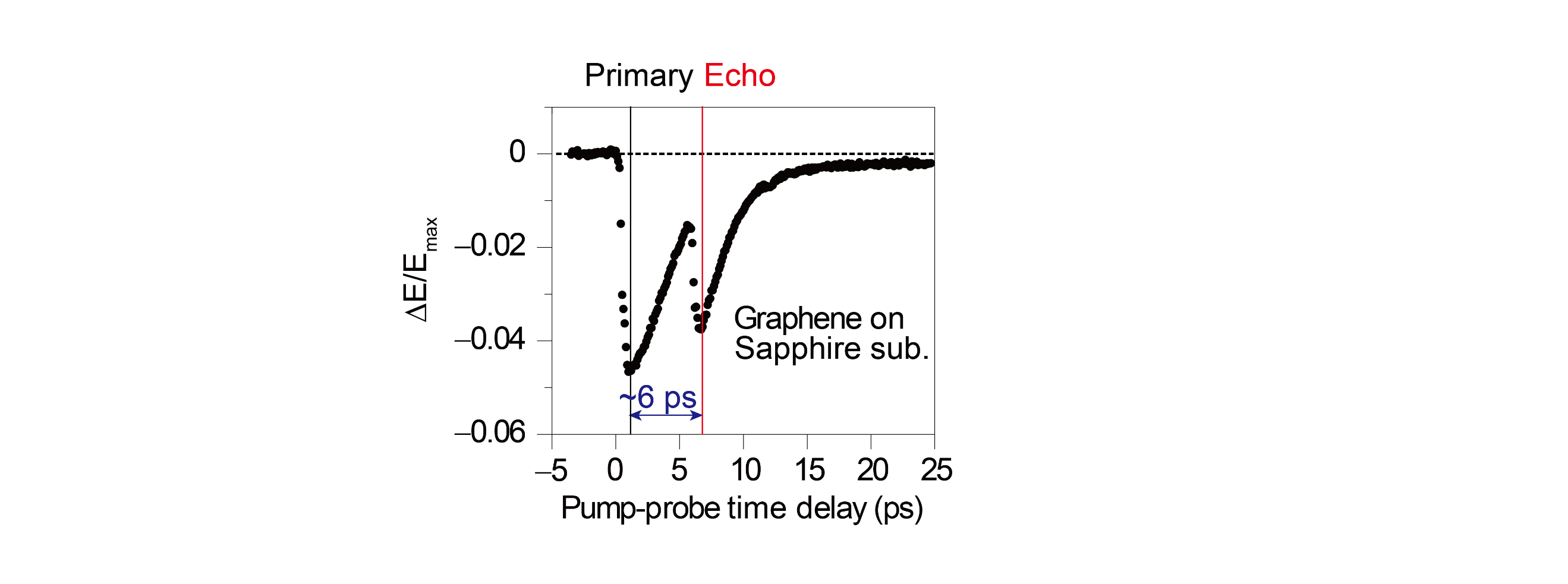
**

**Supporting Fig. S4. Substrate-induced echo signal. A graphene sample deposited on a sapphire substrate with ~430 μm thickness (identical to that used for Bi_2_Se_3_ film growth) was employed for this measurement.**

**
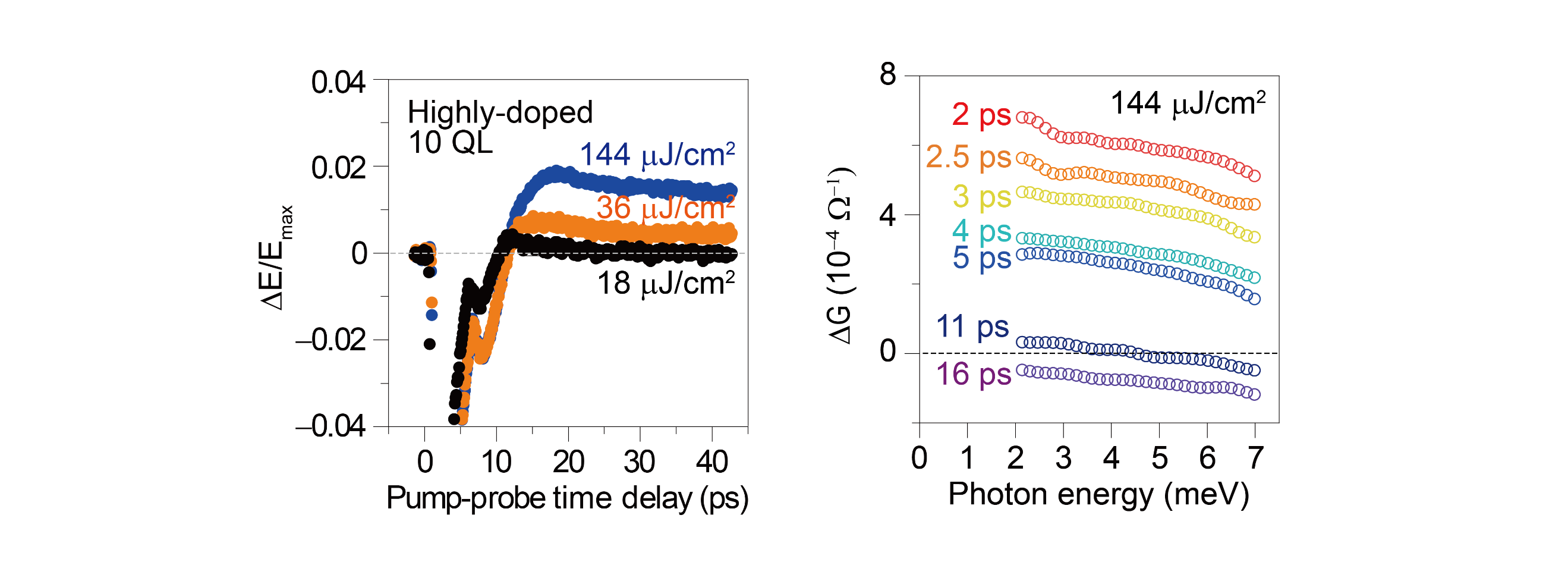
**

**Supporting Fig. S5. Ultrafast THz dynamics of highly-doped 10-QL Bi_2_Se_3_ film. a**, Confirmation of Phase II presence in a highly doped 10-QL sample. The ΔE/E_max_ data is shown for various optical pump fluences (18, 36, and 144 µJ/cm²), demonstration the fluence-dependent transition to Phase II. **b**, Presence of negative ΔG at 11 ps after optical pumping with a fluence of 144 µJ/cm². The gradual decrease of ΔG after 2 ps resembles the dynamics observed in the lowly-doped 10-QL sample (see Fig. 3b). The strong coupling between the TSS and bulk states, induced by the high chemical potential, results in a merged single Drude peak characterized by an intermediate scattering time.


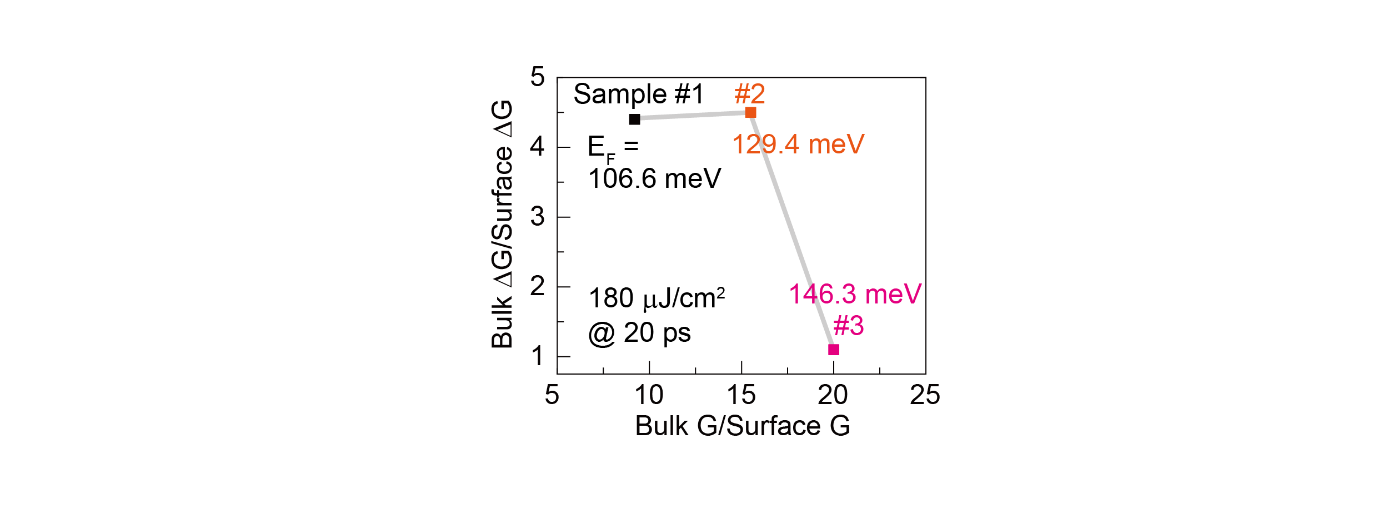
**Supporting Fig. S6. Doping dependence of electron pathways in topological insulators.** A significant reduction in the Bulk ΔG / Surface ΔG ratio occurs when Bulk G / Surface G ≈ 20, likely due to coupling between surface and bulk states, which is commonly observed in highly doped Bi₂Se₃ samples.


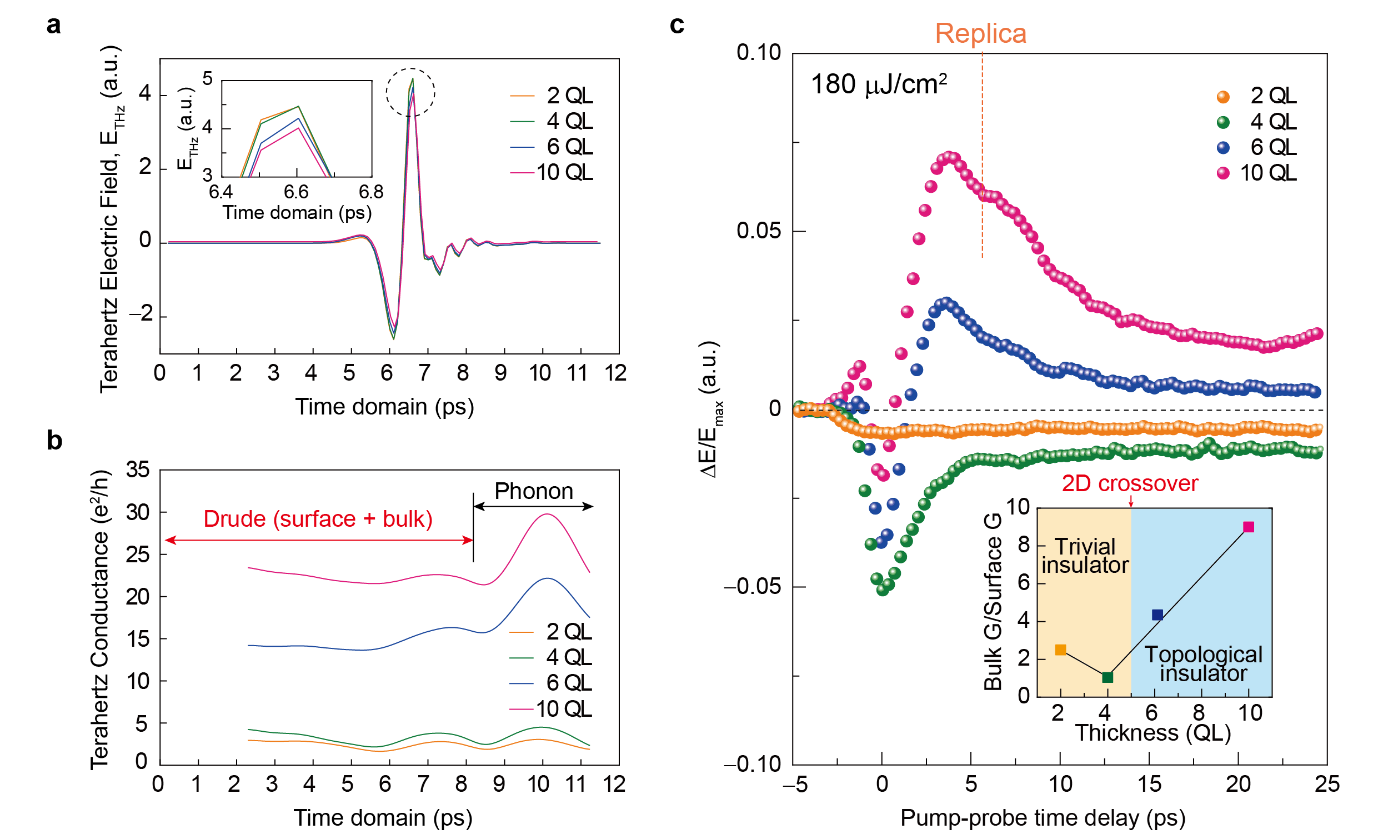
**Supporting Fig. S7. Thickness-dependent electron pathways in topological insulators.** **a**, Time-domain terahertz signals for a series of Bi₂Se₃ thin films. **b**, Corresponding terahertz conductance spectra calculated for the Bi₂Se₃ thin films. **c**, Optical pump–terahertz probe responses (ΔE/Eₘₐₓ) for the Bi₂Se₃ thin films. Inset: Equilibrium phase diagram of Bi₂Se₃ thin films showing the ratio of bulk to surface conductance. Below 5 QL (the 2D crossover), the system transitions from a topological insulator (with interface) to a trivial insulator (without interface). A clear correlation emerges between the equilibrium phase and the nonequilibrium photoexcited state—either topological insulator or excitonic topological insulator—highlighting the critical role of the bulk–surface interface in driving the transition to the excitonic topological insulator phase.

|  | **Drude I for  Bulk** | **Drude II  for TSS** |
| --- | --- | --- |
| **ω_p_ (plasma frequency) [cm^-1^]** | 3922.2286 | 548.27882 |
| **γ (scattering rate) [cm^-1^]** | 391.70417 | 14.99996 |

|  | **Lorentz for Phonon** |
| --- | --- |
| **Ω_0_ (center frequency)[cm^-1^]** | 68.76972 |
| **Ω_p_ (oscillator strength)[cm^-1^]** | 430.85681 |
| **Г (broadening factor)[cm^-1^]** | 9.06018 |

**Supporting Table 1.** Drude-Lorentz model parameters for the 10-QL Bi_2_Se_3_ film in equilibrium.

| **Feature** | **Conventional Photodoping** | **Pathway-Selective Mechanism (Ours)** |
| --- | --- | --- |
| **Driving factor** | Change in total carrier number (valence → conduction band) | Band alignment at surface–bulk interface enforcing pathway selectivity |
| **Mechanism of phase transition** | Fermi level shift, screening change | Directionality and coherence of electron motion along distinct orbital/momentum channels |
| **Role of carrier density** | Primary | Secondary / not decisive |
| **Nature of transition** | Density-driven, similar to metal–insulator transition | Topology-enabled, coherence-driven, collective phase |
| **Experimental signature** | Can be reproduced by varying carrier number alone | Requires specific excitation pathways; cannot be reproduced by carrier number change alone |
| **Novelty** | Well-established in semiconductors and correlated systems | Demonstrates a fundamentally new, pathway-selective phase transition in a topological system |

**Supporting Table 2. Comparison of our path-selective principle with conventional photodoping.**
